# The Use of the Dynamics of Changes in Table Eggs during Storage to Predict the Age of Eggs Based on Selected Quality Traits

**DOI:** 10.3390/ani11113192

**Published:** 2021-11-09

**Authors:** Kamil Drabik, Tomasz Próchniak, Kornel Kasperek, Justyna Batkowska

**Affiliations:** Institute of Biological Basis of Animal Production, University of Life Sciences in Lublin, 13 Akademicka St., 20-950 Lublin, Poland; kamil.drabik@up.lublin.pl (K.D.); kornel.kasperek@up.lublin.pl (K.K.); justyna.batkowska@up.lublin.pl (J.B.)

**Keywords:** egg storage, time, egg weight class, statistical models, regression

## Abstract

**Simple Summary:**

The freshness is the most important characteristic of table eggs. EU legislation does not provide clear guidelines how to store table eggs or how to elongate their shelf life. Changes occurring in eggs after laying are a natural consequence of the passage of time, and there is no method for precise determination of “age” in a randomly chosen egg. The dynamics of changes of individual quality features of the raw material during its extended storage period of up to 35 days were determined. For this purpose, the evaluation of quality traits was performed daily, and the data thus obtained made it possible to create a multivariate mathematical model which, after further statistical processing, makes it possible to determine with high certainty (above 95%) the age of an egg on the basis of its measurable traits, both non-destructive and destructive. The study allowed us to select easily measurable egg quality traits, whose values clearly change in time. The detailed data of daily variability and methods of data statistical analysis are not only of scientific importance, but are also a useful diagnostic tool in assessing the freshness of table eggs on the basis of their quality characteristics.

**Abstract:**

The aim of the study was to determine daily changes in some egg quality parameters, indirectly reflecting egg freshness, and to assess the possibility of predicting time from laying using mathematical methods. The study material consisted of 365 table eggs of medium (M, ≥53 g and <63 g) and large (L, ≥63 g and <73 g) weight classes (commercial stock, cage system, brown-shelled eggs) collected on the same day. Eggs were numbered individually and placed on transport trays and stored (14 °C, 70% RH). Every day, for 35 days, egg quality characteristics were analyzed (10 eggs per group). The change of traits in time was analyzed on the basis of linear and polynomial regression equations, depending on the trait. Based on model fitting, eight traits were selected as those most affected by storage time: egg weight and specific weight, Haugh units, albumen weight, air cell depth, yolk index, albumen and yolk pH. These traits, excluding those related to the weight, were then used in a multiple linear regression model to predict egg age. All regression models presented in this study were characterized by high predictive efficiency, which was confirmed by comparison of the observed and estimated values.

## 1. Introduction

Quality of table eggs is often studied. It is influenced by a number of factors related to bird origin, feeding, flock age or rearing system. However, regardless of them, soon after laying, biophysical and chemical changes take place in eggs, which have negative influence on egg quality.

The storage of table eggs in the EU is regulated by the Commission Regulation (EC) No 589/2008 [1]. The important element described in the Regulation is minimal shelf life date defined at 28 days and quality parameters to be met by eggs classified as Class A. In terms of storage, the most important of them is the air cell depth, for which the limit has been adopted as 6 mm for eggs classified as an Class A.

At the same time, the air cell is not the only feature that changes with egg storage time. The primary and most easily observable trait is egg weight loss [2,3]. The change in egg mass occurs primarily through evaporation of water from the egg contents via gas exchange that occurs between the egg contents and the external environment [4]. Due to the fact that weight loss during storage occurs regardless of weight grade or storage conditions, it may cause a number of problems for distributors and producers of table eggs. In the case of eggs with weights close to the lower limits of weight classes specified in the Regulation, the weight may decrease during the trade, and this change, despite its natural causes, is considered an adulteration (mislabeling).

Specific morphological elements of the egg are also affected by the quality changes. There is a thinning of the albumen dense fraction, and a change in its mass and pH [5,6]. Similar changes also occur in the yolk, which, due to the diffusion of water from the albumen [7], increases its mass while its shape index value decreases. Similar to the albumen, the yolk pH also tends to increase with the storage time.

In the aspect of storage, it is necessary to discuss the factors that can affect the intensity of changes in raw material. One of the most important is temperature, while its reduction has an inhibitory effect on the intensity of the occurring changes [8,9]. No less relevant in this respect is relative humidity, as providing it at high levels reduces water evaporation from the egg content, inhibiting other changes in egg quality [10].

Recently, the alternative methods of egg protection against negative changes in their quality during the storage have also gained popularity. Among them, it is possible to separate two main groups: those using modification of the atmosphere in the egg environment [11,12] or those using shell pore sealers [13,14,15].

The application of protection treatments or lowering the temperature affects the inhibition of the intensity of changes, but does not inhibit them completely. Moreover, although the bird rearing system or the flock age determine the quality of the egg material, the general trends occurring irrespective of the egg origin are evident for storage [16,17].

Therefore, these observations indicate a natural origin of the mentioned variability. Unfortunately, the majority of works dealing with changes in the quality of table eggs during storage focus on specific periods, usually 7 days long. This allows the analysis of the influence of time on external and internal characteristics of eggs, but makes it impossible to evaluate daily changes of their quality. Therefore, it seems reasonable to analyze their dynamics, which would enable bidirectional analysis, i.e., changes in the egg material quality on particular days, or vice versa, i.e., it would allow determination of the age of an egg on the basis of its quality characteristics. Determination of daily variability will not only allow for detailed observations, which may contribute to better understanding of the process of egg material quality deterioration, but also be used as a diagnostic tool in its assessment. In the case of eggs remaining on the market, for example, quality control mainly consists of comparing the weight declared by the producer with the actual situation. Therefore, non-compliance is possible. The use of the model can therefore verify whether the indicated inconsistencies are due to incorrect classification of the raw material or are related to its natural variability during storage. At present, the characteristics laid down in the Regulation remain the basis for this, while others are omitted. The identification of traits whose variability over time shows a stable tendency to change may resolve the problem of the hitherto inadequate assessment of material and eliminate erroneous conclusions regarding potential adulteration of the marketed eggs.

The aim of the study was to determine daily changes in some egg quality parameters, indirectly reflecting freshness, and to assess the possibility of predicting time from laying using mathematical methods.

## 2. Materials and Methods

### 2.1. Eggs

A total of 730 table hen eggs collected on the same day, 365 eggs each of medium (M, ≥53 g and <63 g) and large (L, ≥63 g and <73 g) weight classes. The eggs were purchased from a farm keeping a commercial flock of caged brown-shelled egg layers. Birds were kept in accordance with the requirements of Council Directive 1999/74/EC of 19 July 1999 laying down minimum standards for the protection of laying hens. The stocking density was 750 cm^2^/bird (5–6 birds per cage). Additionally, cages were equipped with a group nest, litter, perches (15 cm per laying hen) and claw-shortening devices. Birds were maintained according to zoo-hygienic and welfare requirements. All birds were fed with the same complete feed for laying hens, suitable for the age of the birds and laying phase (ME 11.6 MJ, protein 17.0%, fiber 3.9%, Ca 3.8%, P 0.55%).

The choice of material was determined by the facts that the typical consumer usually chooses eggs from these two classes, as the most common on the market, they generally come from cage farming and the brown eggshell is preferred.

### 2.2. Egg Quality Analyses

Eggs were individually numbered and placed blunt end up on transport carton trays, then stored at 14 °C and 70% humidity (typical storage conditions). On the day of starting the experiment (day 0), egg quality characteristics were assessed (15 eggs each), then the same testing procedures were repeated by the same laboratory team for the next 35 days (10 eggs per group daily). Additionally, every 7 days all the eggs included in the experiment were weighed. The Instron Mini 55 (Instron^®^, Norwood, MA, USA) compression apparatus and the TSS^®^ Egg Quality Measurement (EQM, TSS^®^, York, UK) analytical kit were used. The characteristics of the whole egg were analyzed as follows:

- egg shape index (EI, as a ratio of its width and length, using electronic caliper),
- air cell depth (ACD, by candling, according to scale),
- egg weight (EW, using laboratory balance with 0.01 g accuracy),
- egg specific gravity (SG, based on egg weight measurement in the air and in the water, according to Archimedes principle),
- proportions of particular egg elements (as the ratio of their weight to the weight of whole egg).

In the case of eggshell quality, the following traits of this element were evaluated:

- color (SC, as a percentage of reflected light),
- weight (SW, using laboratory balance with 0.01 g accuracy),
- thickness (ST, by micrometer screw, at the “equator”),
- density (SD, calculated based on shell area and volume, according to Shafey [18].

In the case of egg content, the height of dense albumen (AH) was measured, and based on it the Haugh units (HU) were calculated [19]. As far as the yolk traits are concerned, its weight (YW, using laboratory balance with 0.01 g accuracy), color (YC, using 16-point Roche scale, DSM^®^) and index (YI, as a ratio of height and diameter) were estimated. The pH of albumen (ApH) and yolk (YpH) were measured using a pH meter with a combined glass electrode.

### 2.3. Statistical Analyses

The following statistical analyses were used: CORR, REG, TTEST, GLM procedures of SAS software (Statistical Analysis System, 9.4, Cary, NC, USA, 2013). The effect of storage time on trait values is presented as Spearman’s rank correlations and regression equations. Regression models depending on the trait analyzed were selected on the basis of graphical analysis of residuals and the value of the R^2^ coefficient. Consequently, egg weight and specific weight were analyzed by linear regression models (Model 1), while for Haugh units, albumen weight, air cell depth, yolk index and albumen and yolk pH, a polynomial of the third degree regression was used (Model 2).

$$\mathrm{Model}\;1:\;{Y=\beta }_{0}{+\beta }_{1}*{\mathrm{day}+\varepsilon }_{i}$$

$$\mathrm{Model}\;2:\;{Y=\beta }_{0}+\beta_{1}*\mathrm{day}+\;\beta_{2}*{\mathrm{day}}^{2}+\beta_{3}*{\mathrm{day}}^{3}+\varepsilon_{i}$$

where:

Y—estimated value of egg trait
$\beta_{0}$—intercept
$\beta_{1}{,\;\beta }_{2}{,\;\beta }_{3}$—coefficient of the polynomial term

A multivariate linear regression model (model 3) was used to predict egg storage day. Weight-related traits were dropped from the model to eliminate the influence of egg weight class. Consequently, estimation was based on SG, ApH, YpH, YI, HU and ACD. No high correlations (0.8 and above) were found between the traits used in the storage day prediction model. The lack of multicollinearity between traits was also confirmed through the variance inflation factor and tolerance. Model fit, with successively added traits, was presented using R^2^ and Mallows’ statistic C(p).

$$\mathrm{Model}\;3:\;{Y=\beta }_{0}{+\beta }_{1}*{\mathrm{SG}+\beta }_{2}*{\mathrm{ApH}+\beta }_{3}*{\mathrm{YpH}+\beta }_{4}*{\mathrm{YI}+\beta }_{5}*{\mathrm{HU}+\beta }_{6}*{\mathrm{ACD}+\varepsilon }_{i}$$

where:

Y—estimated value of egg trait
$\beta_{0}$—intercept
*β*_1_–*β*_6_—coefficient of the polynomial term

The verification of the presented models (Model 1, 2, 3) was performed by estimating the trait values and then comparing the estimated values with the expected ones using Student’s *t*-test for combined variables. For Models 1 and 2, trait values were predicted based on the day of storage. For Model 3, the day of storage was predicted from the trait values.

The Supplementary Material presents basic statistics of the analyzed traits including the significance of differences verified by two-factor analysis of variance with Tukey’s test (proc GLM). Egg weight classes (2 levels) and day of egg storage in a 7-day period (6 levels) were used as factors.

## 3. Results

### 3.1. Dynamics of Egg Quality Changes during Storage

Only those traits for which the regression models had an R^2^ > 0.1 (EW, SG, ACD, HU, AW, ApH, YI, YpH) are presented in the results. The complete data are included as Supplementary Materials (Table S1).

Independently of the weight class, Spearman correlation analysis showed a significant effect of storage time on the value of traits, except for YW (Table 1). It was found that the value of characteristics such as egg weight, specific weight, albumen (HU, AH, AW) and yolk features (YI, YC) decreased significantly with time. On the other hand, the air cell depth as well as the albumen and yolk pH were positively correlated with the storage time.

**Figure 1:**
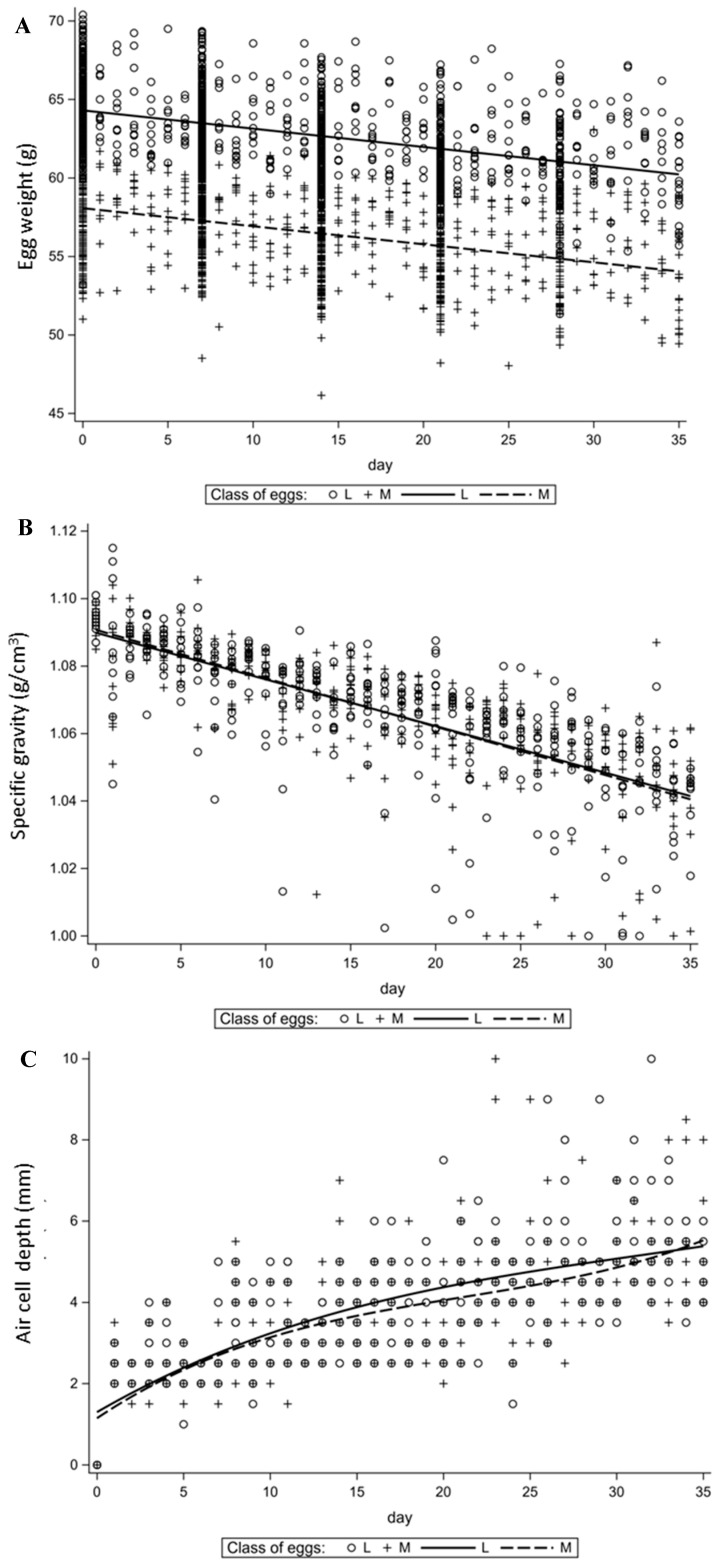
Changes in time of selected traits of whole egg. (**A**)—egg weight (EW), (**B**)—specific gravity (SG), (**C**)—air cell depth (ACD).

In terms of egg weight and its specific weight, a linear relationship with the egg material storage time was found (Figure 1A,B), while for the other egg quality traits non-linear relationships were found, so their analysis was based on polynomial regression models. For SG, a small scatter of results from the simple regression was found, characterized by a high fit (R^2^ = 0.53). The occurrence of outliers in this range is due to the deepening of the air cell, which in some cases caused the inability to analyze the wet egg weight, which resulted in a value of ≈1.0. When comparing the increase in air cell depth, an identical relationship was noted within both weight classes up to day 10 of the experiment. After this time, the tendency to a higher rate of ACD deepening was characterized by L class eggs.

**Figure 2:**
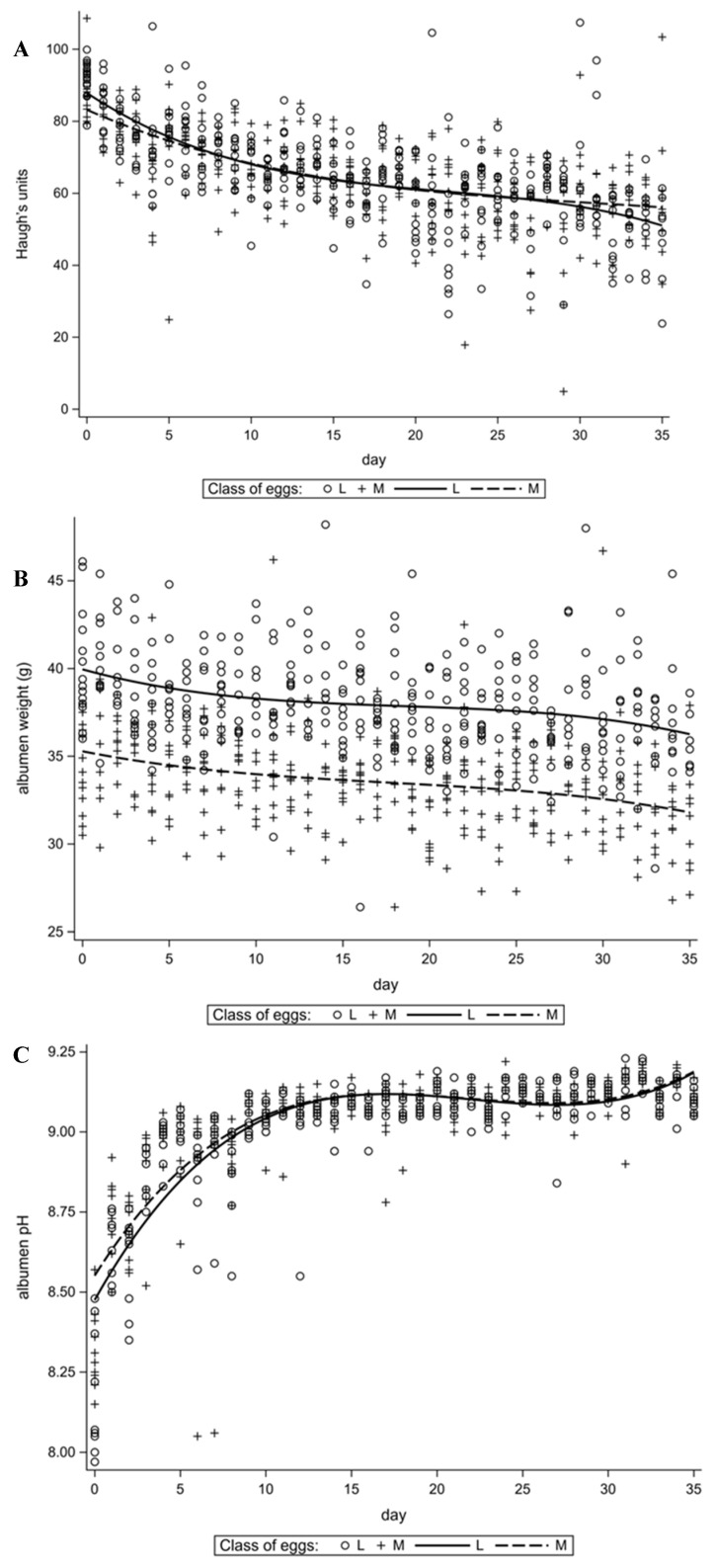
Changes in time of selected traits of egg albumen. (**A**)—Haugh units (HU), (**B**)—albumen weight (AW), (**C**)—albumen pH (ApH).

Analysis of albumen quality traits (Figure 2) showed a small difference according to weight class for HU (Figure 2A). After 5 days of the experiment, these differences were imperceptible. An interesting observation concerns albumen pH (Figure 2C). This trait was characterized by fluctuating variability during storage. An increase in pH was observed up to about the 15th day of the experiment, with the highest intensity of changes observed in the initial period (10th day) of the experiment. In later stages of the study (up to the 30th day), a slight decrease in the trait value was observed. It is worth mentioning that ApH was the trait with the highest fitting index to the regression equation.

**Figure 3:**
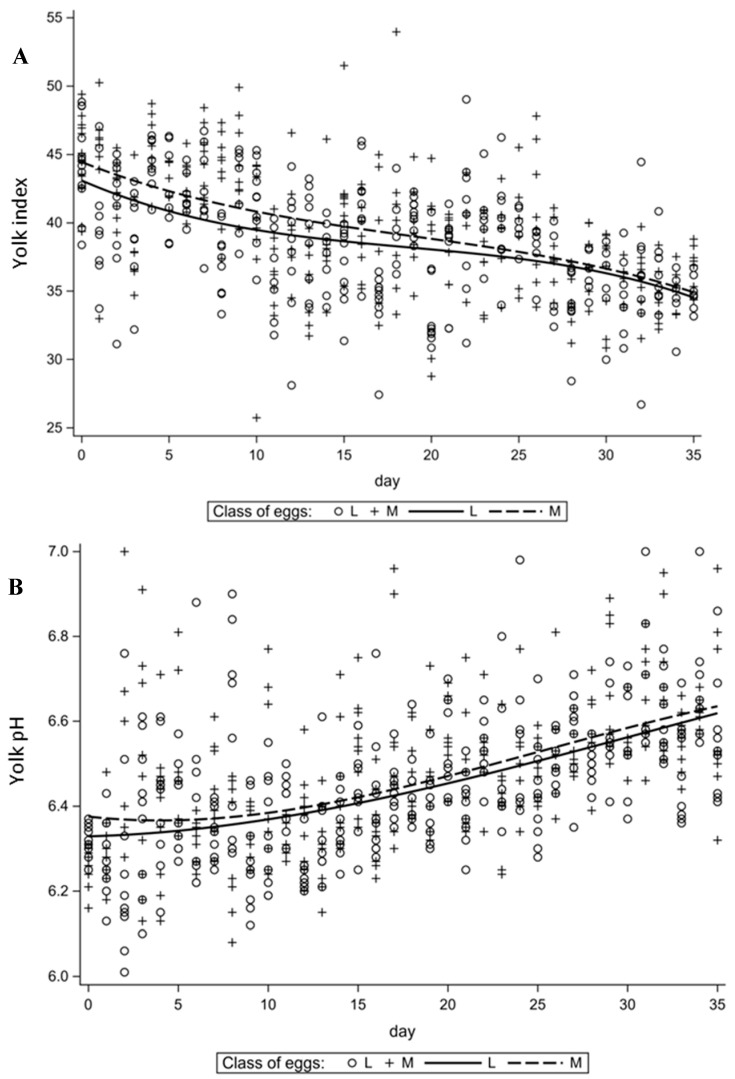
Changes in time of selected traits of egg yolk. (**A**)—yolk index (YI), (**B**)—yolk pH (YpH).

In the case of the yolk quality characteristics (Figure 3), a decrease in the yolk index value was observed with storage time regardless of egg weight class. In the yolk pH (Figure 3B), in contrast to egg albumen, initially a slight increase in the value of the trait (up to the 10th day of the experiment) was found, after which the increase continued more intensively until the end of the experiment.

Each of the models used, regardless of the trait analyzed, was characterized by high significance (Table 2). The fit of the data to the model (R^2^) ranged from 0.1 for albumen weight in L class eggs to 0.71 for ApH of eggs of the same weight class. High R^2^ values were also found for traits such as SG and ACD, regardless of the weight class of the eggs analyzed. The lowest values of the R^2^ coefficient related to egg weight (0.17 and 0.2 for M and L grade eggs, respectively) and albumen weight (0.11 and 0.1 for M and L grade eggs, respectively). Student’s *t*-tests comparing observed and predicted variables based on regression equations show no significant differences, confirming good model fitting.

### 3.2. Prediction of Egg Age based on the Dynamics of Changes in Egg Quality

The prediction of egg age based on the six trait values resulted in a model with a high fit index (R^2^ = 0.72) (Table 3). Moreover, Mallows’ statistic C(p) is below the degrees of freedom of the model. This also means a very good fitting of the model in terms of this criterion. Each of the characteristics used in the model showed highly significant influence on the model solution. The difference between the model predicted and observed egg storage days was not significant.

The residuals obtained from the regression model were assessed for lack of autocorrelation, their normal distribution and heteroscedasticity. The results obtained indicate that the assumptions of the regression analysis are met and the model fits the numerical data.

## 4. Discussion

The prediction of egg age based on the analysis of its quality traits has been the subject of several works, varied by the measurement techniques used, as well as the traits that are involved in model construction. Thus far, analyses of this type have included S-ovoalbumine content [20], or albumen pH and Haugh units [21,22]. It seems that a more accurate prediction can be achieved by using not one, but at least several, features in the model, so perhaps the solution proposed in our study is characterized by a more comprehensive approach. Moreover, the authors of the mentioned works assumed a linear variation of traits during egg storage, while our study clearly shows a non-linear character of most of them.

Attempts have been made to use various methods to predict the magnitude of egg quality changes. Ragni et al. [23] used a technique for this purpose based on the dielectric properties of eggs and the variability of parameters during the storage period. The authors used the data obtained subsequently to determine a linear regression for changes in raw material quality, similar to that developed in their own study. A similar analytical method was also used by Soltani et al. [24], who additionally used neural networks to analyze the data obtained. Analyses performed using electronic nose and chemometric methods were also used to predict egg quality traits, including yolk index [25]. At the same time, the authors performed data analyses based on neural networks, which allowed determination of a model for changes in yolk index. An interesting method for analyzing eggs to predict their age was also proposed by Tan et al. [26], who analyzed changes in the center of gravity of eggs during storage using magnetic resonance. Based on the collected data, a model was developed to predict the age of eggs, which was characterized by a high fitting (R^2^ = 0.961). The indicated solutions, although effective and reliable, required the use of specialized equipment. Therefore, despite considerable methodological advancement, it seems that practical use of the results obtained may be difficult. In our study, data were obtained using a minimum of available laboratory tools, giving a real chance for the models presented to be used in practice. From the model predicting the day of storage, measures related to the egg weight have been removed, making it more universal for different weight classes of eggs.

Changes in egg quality during their storage are influenced by factors affecting the laying hens. In order to be able to standardize the obtained results, it was necessary to unify the raw material itself. The study of Batkowska et al. [27] indicates that the intensity of quality changes in eggs with time after the oviposition may depend not only on the housing system of the birds, but also on the weight class of the eggs themselves. Therefore, two of the most popular egg weight classes on the EU market were chosen for the study. This is also related to consumer preferences, whose egg weight choices are driven by many factors, but in many countries, higher weight eggs are preferred [28]. At the same time, the final Model 3 proposed in our study omits egg weight, making it universal in this respect.

The egg weight loss and the related changes in specific gravity are among the most frequently reported observations in terms of storage studies of raw egg material regardless of its origin or storage conditions [7,29]. Weight loss occurs by evaporation of water through the shell pores, which are not uniform in number, size or distribution on the surface [30]. The shell pores play an important role in the incubation process, but in terms of table eggs, the porosity may affect the shelf life of the raw material. This is because excessive permeability and/or pore number increases egg weight loss through evaporation [31].

Many authors also identify the role of the most external layer of the egg, the cuticle, in the gas exchange process. The mucin layer protects eggs from microbial contamination and increases shell strength [32]. Although the effective protective function of the cuticle is only a few days [33], the need to keep it intact has restricted the washing of table eggs in EU countries [1]. In our study, weight loss was observed but there was no acceleration in its rate, which could only be indicated by the temporary protection of the eggs provided by the mucin layer. Furthermore, egg weight and specific weight were the only linear variables. On the other hand, Liu et al. [34] analyzed the effect of egg washing on their quality during storage, taking into consideration the cuticle covering of the shell. Their results indicate a faster deepening of the air cell in washed eggs, which may confirm the protective effect of the mucin layer.

Egg albumen also deteriorates during its storage. The most important factors include a drop in the Haugh units, a universal unit which takes into account the weight and height of the albumen. Our own research has shown a non-linear decrease in this value. The decrease in HU has also been noted by other authors [10,35]. Water loss, both through evaporation and due to its migration from the albumen to the yolk, is claimed to be the main factor influencing the change in dense albumen structure [36]. Additionally, other factors such as reduction of disulfide bonds or ovomucin depolymerization caused by alkaline hydrolysis are also indicated. The breakdown of bonds stabilizing the ovomucin–lysozyme complex is an important factor in this aspect [37]. A change in the structure of the mentioned complex contributes to the dilution of the albumen, and it has been found that the interaction between these two egg components is significantly weakened with increasing pH [38].

The change in albumen pH during storage is caused by the release of carbon dioxide through the shell pores, which contributes to the alkalization of the egg contents [39]. However, in our study it was found that the change in albumen pH is not linear. Underlying this relationship is the buffering capacity of the albumen itself. Studies of Heath [36] have shown that egg albumen has a buffering capacity based on a carbonate buffer. The highest buffering capacity was recorded for pH not exceeding 8. Increasing the pH decreased the buffering capacity of the albumen. These observations seem to be in line with those found in our study, especially since the pH recorded exceeded the values indicated in the cited work as the limit for the best buffering capacity of the albumen.

The changes in yolk quality parameters are mainly related to the movement of water between the particular morphological elements of the egg. It should be noted that this movement is mainly related to the penetration of water through the vitelline membrane from the albumen to the yolk [40]. One of the noticeable effects of water movement is the decrease in yolk shape index values observed by many authors, as well as in the strength of the vitelline membrane [10,17], which, in case of prolonged storage time, may lead to its rupture and the mixing of egg content elements. Our own research is consistent with the trend described, but it should be noted that these changes are not linear, as in the case of albumen traits, which suggests the presence of a relationship between the indicated egg quality traits.

## 5. Conclusions

The study showed that only two of the analyzed egg quality traits (egg weight and specific weight) showed linear variability during storage. The change in values of traits such as air cell depth, albumen height and Haugh units, albumen and yolk pH and yolk index during the storage of eggs should be analyzed non-linearly, as indicated by high fit indices of regression equations.

The first part of the study allowed us to select easily measurable egg quality traits, whose values clearly change over time. The use of these traits, excluding those related to weight, resulted in a highly fitted multivariate predictive model for determining the age of eggs. The validity of all regression models used in this study was confirmed by estimating the expected values and comparing them to the real ones. The model is applicable when typical egg storage conditions are used, as in our work, but the influence of temperature on the dynamics of egg quality changes requires further research to determine temperature-dependent models.

## Figures and Tables

**Table 1:** Spearman’s correlation coefficients between particular quality traits of eggs and days of storage.

Class	M		L	
Trait	ρ	*p*-Value	ρ	*p*-Value
EW	−0.20287	0.0001	−0.20407	0.0001
SG	−0.82275	<0.0001	−0.79993	<0.0001
ACD	0.76409	<0.0001	0.75573	<0.0001
HU	−0.62356	<0.0001	−0.69372	<0.0001
AW	−0.35078	<0.0001	−0.32698	<0.0001
ApH	0.73197	<0.0001	0.77513	<0.0001
YI	−0.59984	<0.0001	−0.50617	<0.0001
YpH	0.38091	<0.0001	0.38466	<0.0001

EW—egg weight, SG—egg specific gravity, ACD—air cell depth, HU—Haugh units, AW—albumen weight, ApH—albumen pH, YI—yolk index, YpH—yolk pH.

**Table 2:** Statistical models used for particular trait analysis.

Trait	EWC	Model	Pr > F	R^2^	MD (Observed – Expected)	SD	Pr > T	Model Type
EW	M	EW = 58.06968 − 0.11497 ∗ day	<0.0001	0.17	−0.00001	2.496	0.9999	linear
	L	EW = 64.30016 − 0.11649 ∗ day	<0.0001	0.20	−0.00002	2.285	0.9997	
SG	M	SG = 1.09072 − 0.00144 ∗ day	<0.0001	0.53	0.000082	0.014	0.9124	linear
	L	SG = 1.08987 − 0.00138 ∗ day	<0.0001	0.53	−0.00006	0.014	0.9353	
ACD	M	ACD = 1.14811 + 0.28495 ∗ day − 0.01024 ∗ day^2^ + 0.00016181 ∗ day^3^	<0.0001	0.54	0.00179	1.072	0.9749	polynominal
	L	ACD = 1.29760 + 0.24602 ∗ day − 0.00586 ∗ day^2^ + 0.00006181 ∗ day^3^	<0.0001	0.56	−0.00103	1.062	0.9854	
HU	M	HU = 83.17175 − 1.99460 ∗ day + 0.05648 ∗ day^2^ − 0.00061806 ∗ day^3^	<0.0001	0.37	0.00126	10.460	0.9982	polynominal
	L	HU = 87.84322 − 3.00134 ∗ day + 0.12044 ∗ day^2^ − 0.00185 ∗ day^3^	<0.0001	0.46	−0.00045	10.330	0.9993	
AW	M	AW = 35.27816 − 0.19537 ∗ day + 0.00798 ∗ day^2^ −0.00014972 ∗ day^3^	<0.0001	0.11	−0.00067	2.516	0.9961	polynominal
	L	AW = 39.94734 − 0.27154 ∗ day + 0.01282 ∗ day^2^ − 0.0002307 ∗ day^3^	<0.0001	0.10	−0.00161	2.669	0.9911	
ApH	M	ApH= 8.55088 + 0.08458 ∗ day − 0.00407 ∗ day^2^ + 0.00006202 ∗ day^3^	<0.0001	0.64	0.00112	0.115	0.8581	polynominal
	L	ApH= 8.47459 + 0.09553 ∗ day − 0.00457 ∗ day^2^ + 0.00006905 ∗ day^3^	<0.0001	0.71	0.00156	0.110	0.7922	
YI	M	YI = 44.44111 − 0.50472 ∗ day + 0.01726 ∗ day^2^ − 0.00030335 ∗ day^3^	<0.0001	0.32	−0.0007	3.768	0.9975	polynominal
	L	YI= 43.08972 − 0.55522 ∗ day + 0.02352 ∗ day^2^ − 0.00041845 ∗ day^3^	<0.0001	0.25	0.000812	3.674	0.997	
YpH	M	YpH= 6.37524 − 0.00472 ∗ day + 0.00064422 ∗ day^2^ − 0.00000850 ∗ day^3^	<0.0001	0.27	−0.00000718	0.145	0.9993	polynominal
	L	YpH= 6.32879 + 0.00106 ∗ day + 0.00032864 ∗ day^2^ − 0.00000350 ∗ day^3^	<0.0001	0.31	−0.00003	0.135	0.9962	

EWC—egg weight class, EW—egg weight, SG—egg specific gravity, ACD—air cell depth, HU—Haugh units, AW—albumen weight, ApH—albumen pH, YI—yolk index, YpH—yolk pH, Pr > F—regression model significance, R^2^—determination coefficient, MD—mean of differences (real value – expected value), SD—standard deviation of differences, Pr > T—significance of *t*-test for dependent samples.

**Table 3:** Statistical model used to predict the age of the eggs (in days).

Step	Variable Entered	Partial R^2^	Model R^2^	C(p)	Parameter Estimate	Pr > |t|
Intercept					17.90251	0.5232
1	SG	0.5176	0.5176	382.072	−152.426	<0.0001
2	ApH	0.1096	0.6272	175.732	10.63105	<0.0001
3	YpH	0.0447	0.6719	92.7676	12.82286	<0.0001
4	YI	0.0206	0.6926	55.5100	−0.33684	<0.0001
5	HU	0.0182	0.7108	22.8532	−0.12071	<0.0001
6	ACD	0.0094	0.7202	5.87	1.11087	<0.0001

Paired *t*-test for day * estimated day: mean difference = −0.00031; SD = 5.49; Pr. > |t| = 0.999; SG—specific gravity, ApH—albumen pH, YpH—yolk pH, YI—yolk index, HU—Haugh units, ACD—air cell depth, mean different = day –estimated day.

## Data Availability

The data presented in this study are available on request from the corresponding author.

## Supplementary Materials

The following are available online at https://www.mdpi.com/article/10.3390/ani11113192/s1, Table S1: The mean values of particular traits evaluated during the egg storage depending on the egg weight class and the time of storage.
