# Supplementary material for: The Use of the Dynamics of Changes in Table Eggs during Storage to Predict the Age of Eggs Based on Selected Quality Traits
Source: Animals (Basel). 2021 Nov 9;11(11):3192. doi: 10.3390/ani11113192 (PMC8614337; doi:10.3390/ani11113192)
Supplement: Supplementary file 1 [file animals-11-03192-s001.zip › animals-1450186-supplementary.pdf]

**Table S1**. The mean values of particular traits evaluated during the eggs storage depending on the egg weight class and the time of storage

| Trait | Weight Class (WC) | Day (D) 0 | 7 | 14 | 21 | 28 | 35 | SEM | Factor WC | D | WC×D |
|---|---|---|---|---|---|---|---|---|---|---|---|
| Air cell depth (ACD, mm) | M | - | 2.40 $^{c}$ | 4.55 $^{ab}$ | 4.65 $^{ab}$ | 4.83 $^{ab}$ | 5.11 $^{a}$ | 0.283 | 0.103 | <0.001 | 0.175 |
| | L | - | 2.85 $^{c}$ | 3.61 $^{bc}$ | 4.15 $^{ab}$ | 4.72 $^{ab}$ | 4.61 $^{ab}$ | 0.247 | | | |
| Egg weight (EW, g) | M | 56.3 $^{c}$ | 57.7 $^{bc}$ | 55.3 $^{c}$ | 56.3 $^{c}$ | 55.3 $^{c}$ | 54.1 $^{c}$ | 0.377 | <0.001 | 0.028 | 0.722 |
| | L | 62.9 $^{a}$ | 63.4 $^{a}$ | 63.2 $^{a}$ | 61.7 $^{ab}$ | 61.0 $^{ab}$ | 61.1 $^{ab}$ | 0.357 | | | |
| Eggs specific gravity (SG, g/cm$^{3}$) | M | 1.094 $^{a}$ | 1.078 $^{ab}$ | 1.071 $^{bc}$ | 1.055 $^{bcdef}$ | 1.048 $^{def}$ | 1.043 $^{ef}$ | 0.003 | 0.719 | <0.001 | 0.315 |
| | L | 1.095 $^{a}$ | 1.073 $^{bc}$ | 1.065 $^{bcd}$ | 1.062 $^{bcde}$ | 1.058 $^{cdef}$ | 1.042 $^{f}$ | 0.003 | | | |
| Shell proportion in egg weight (SP, %) | M | 13.3 | 12.9 | 13.4 | 13.0 | 13.3 | 13.5 | 0.104 | 0.977 | 0.211 | 0.776 |
| | L | 13.6 | 12.7 | 13.1 | 13.5 | 13.2 | 13.5 | 0.140 | | | |
| Albumen proportion in egg weight (AP, %) | M | 60.1 $^{a}$ | 59.6 $^{ab}$ | 58.6 $^{ab}$ | 58.5 $^{ab}$ | 52.7 $^{b}$ | 57.9 $^{ab}$ | 0.746 | 0.001 | 0.022 | 0.068 |
| | L | 62.2 $^{a}$ | 59.5 $^{ab}$ | 64.9 $^{a}$ | 59.3 $^{ab}$ | 60.8 $^{a}$ | 58.9 $^{ab}$ | 0.576 | | | |
| Yolk proportion in egg weight (YP, %) | M | 26.6 $^{b}$ | 27.6 $^{ab}$ | 28.0 $^{ab}$ | 28.5 $^{ab}$ | 34.0 $^{a}$ | 28.5 $^{ab}$ | 0.741 | 0.002 | 0.029 | 0.119 |
| | L | 24.2 $^{b}$ | 27.8 $^{ab}$ | 23.5 $^{b}$ | 27.3 $^{ab}$ | 25.9 $^{b}$ | 27.6 $^{ab}$ | 0.527 | | | |
| Shell colour (SC, %) | M | 23.0 $^{c}$ | 29.5 $^{abc}$ | 33.1 $^{a}$ | 26.1 $^{abc}$ | 27.7 $^{abc}$ | 27.9 $^{abc}$ | 0.741 | 0.260 | <0.001 | 0.627 |
| | L | 24.7 $^{bc}$ | 27.1 $^{abc}$ | 31.6 $^{ab}$ | 23.4 $^{c}$ | 26.8 $^{abc}$ | 27.6 $^{abc}$ | 0.661 | | | |
| Shell weight (SW, g) | M | 7.67 $^{bcd}$ | 7.45 $^{cd}$ | 7.53 $^{bcd}$ | 7.31 $^{d}$ | 7.38 $^{cd}$ | 7.31 $^{d}$ | 0.068 | <0.001 | 0.079 | 0.811 |
| | L | 8.73 $^{ad}$ | 8.05 $^{abc}$ | 8.31 $^{abc}$ | 8.38 $^{ab}$ | 8.21 $^{abcd}$ | 8.18 $^{abcd}$ | 0.090 | | | |
| Shell thickness (ST, mm) | M | 0.325 $^{a}$ | 0.303 $^{a}$ | 0.340 $^{a}$ | 0.238 $^{ab}$ | 0.303 $^{a}$ | 0.319 $^{a}$ | 0.007 | <0.001 | <0.001 | 0.061 |

| | | | | | | | | | | | |
|---|---|---|---|---|---|---|---|---|---|---|---|
| | L | 0.342 $^{a}$ | 0.311 $^{a}$ | 0.316 $^{a}$ | 0.326 $^{a}$ | 0.337 $^{a}$ | 0.354 $^{a}$ | 0.005 | | | |
| Shell density (SD, g/cm$^{3}$) | M | 3.42 $^{b}$ | 3.51 $^{b}$ | 3.20 $^{b}$ | 4.58 $^{ab}$ | 3.54 $^{b}$ | 3.46 $^{b}$ | 0.09 | 0.007 | <0.001 | 0.062 |
| | L | 3.38 $^{b}$ | 3.47 $^{b}$ | 3.37 $^{b}$ | 3.52 $^{b}$ | 3.29 $^{b}$ | 3.16 $^{b}$ | 0.05 | | | |
| Albumen height (AH, mm) | M | 7.92 $^{a}$ | 5.47 $^{b}$ | 4.70 $^{b}$ | 4.34 $^{bc}$ | 4.29 $^{bc}$ | 4.22 $^{bc}$ | 0.26 | 0.307 | <0.001 | 0.445 |
| | L | 8.73 $^{a}$ | 5.76 $^{b}$ | 5.34 $^{b}$ | 4.69 $^{bc}$ | 4.64 $^{bc}$ | 3.30 $^{c}$ | 0.28 | | | |
| Haugh’s units (HU) | M | 89.0 $^{a}$ | 73.1 $^{b}$ | 66.9 $^{b}$ | 62.5 $^{bc}$ | 63.5 $^{bc}$ | 59.2 $^{bc}$ | 1.904 | 0.496 | <0.001 | 0.294 |
| | L | 92.0 $^{a}$ | 72.4 $^{b}$ | 69.5 $^{b}$ | 60.9 $^{bc}$ | 63.8 $^{bc}$ | 47.8 $^{c}$ | 2.213 | | | |
| Yolk weight (YW, g) | M | 15.3 $^{b}$ | 15.9 $^{ab}$ | 15.7 $^{ab}$ | 16.0 $^{ab}$ | 18.8 $^{a}$ | 15.4 $^{ab}$ | 0.408 | 0.316 | 0.099 | 0.066 |
| | L | 15.5 $^{ab}$ | 17.6 $^{ab}$ | 16.9 $^{ab}$ | 16.9 $^{ab}$ | 16.1 $^{ab}$ | 16.8 $^{ab}$ | 0.165 | | | |
| Yolk colour (YC, pts.) | M | 13.2 $^{ab}$ | 12.4 $^{b}$ | 12.2 $^{b}$ | 12.6 $^{b}$ | 12.7 $^{ab}$ | 12.4 $^{b}$ | 0.130 | 0.055 | <0.001 | 0.143 |
| | L | 13.9 $^{ab}$ | 13.5 $^{ab}$ | 12.7 $^{ab}$ | 12.5 $^{b}$ | 12.4 $^{b}$ | 12.4 $^{b}$ | 0.119 | | | |
| Yolk index (YI) | M | 44.5 $^{a}$ | 44.8 $^{a}$ | 37.8 $^{bc}$ | 38.5 $^{bc}$ | 34.1 $^{c}$ | 36.9 $^{c}$ | 0.673 | 0.112 | <0.001 | 0.714 |
| | L | 44.4 $^{a}$ | 42.3 $^{ab}$ | 37.6 $^{bc}$ | 37.7 $^{bc}$ | 34.1 $^{c}$ | 34.9 $^{c}$ | 0.675 | | | |
| Albumen pH (ApH) | M | 8.32 $^{c}$ | 8.92 $^{b}$ | 9.07 $^{ab}$ | 9.07 $^{ab}$ | 9.09 $^{ab}$ | 9.10 $^{ab}$ | 0.042 | 0.624 | <0.001 | 0.164 |
| | L | 8.17 $^{c}$ | 8.94 $^{ab}$ | 9.05 $^{ab}$ | 9.10 $^{ab}$ | 9.13 $^{a}$ | 9.09 $^{ab}$ | 0.048 | | | |
| Yolk pH (YpH) | M | 6.25 $^{e}$ | 6.40 $^{bcde}$ | 6.42 $^{abcd}$ | 6.46 $^{abcd}$ | 6.59 $^{ab}$ | 6.60 $^{a}$ | 0.024 | 0.122 | <0.001 | 0.315 |
| | L | 6.32 $^{de}$ | 6.34 $^{cde}$ | 6.35 $^{cde}$ | 6.40 $^{bcde}$ | 6.50 $^{abc}$ | 6.56 $^{ab}$ | 0.015 | | | |

$^{a, b, c, d, e, f}$ – differences between means within a given trait are significant at $p \leq 0.05$ (Tukey test)
